# The effect of isotretinoin on insulin resistance and adipocytokine levels in acne vulgaris patients

**DOI:** 10.3906/sag-1806-44

**Published:** 2019-02-11

**Authors:** Gonca SOYUDURU, Esra ÖZSOY ADIŞEN, İlkay KADIOĞLU ÖZER, Burhan A. AKSAKAL

**Affiliations:** 1 VM Medical Park Hospital, Kocaeli Turkey; 2 Department of Dermatology, Faculty of Medicine, Gazi University, Ankara Turkey; 3 Department of Dermatology, Meram Faculty of Medicine, Necmettin Erbakan University, Konya Turkey

**Keywords:** Acne vulgaris, adipocytokine, insulin sensitivity

## Abstract

**Background/aim:**

Recent data draw attention to the effect of body composition, insulin resistance, and adipocytokines to acne vulgaris (AV) development. The aim of this study was to assess the association of AV with insulin resistance and adipocytokine levels and to evaluate the effect of isotretinoin on insulin resistance and adipocytokine levels.

**Materials and methods:**

In 29 AV patients and 29 healthy volunteers, body mass index (BMI) and body fat mass (BFM), lipid, adiponectin, leptin, resistin, retinol binding protein-4 (RBP4), and insulin levels were measured and insulin resistance was assessed by HOMA-IR index in serum samples taken twice from patients before and after isotretinoin treatment.

**Results:**

In AV patients, pretreatment HOMA-IR and adipocytokine levels were not found to correlate with disease severity. With five months of isotretinoin treatment, higher HOMA-IR values ​​were found (P = 0.028). Isotretinoin therapy maintained lower mean resistin levels (P = 0.016), higher mean RBP4 levels (P = 0.040), but not affected the mean adiponectin and leptin levels (P = 0.113, P = 0.125, respectively).

**Conclusions:**

All data suggests that five months of isotretinoin therapy in AV patients causes insulin resistance and the increase in insulin resistance is not dependent on age, BMI, BFM, and lipid levels of these patients.

## 1. Introduction

Acne vulgaris (AV) is a chronic inflammatory multifactorial disease of the pilosebase unit that affects 85% of the population aged 12–24 years (1–4). Characteristic lesions are comedones, inflammatory papules, pustules, and nodules and cysts that can lead to scarring and pigmentation changes (1). AV pathogenesis is multifactorial. Abnormal follicular keratinization, increased production of sebum, *Propionibacterium acnes* (*P. acnes*) colonization and inflammation are implicated (1).

13-cis-retinoic acid (isotretinoin) is a vitamin A (retinol) metabolite that has been used for about 30 years in nodulocystic and other treatment-resistant AV treatment (5). The mechanism of action of isotretinoin, a turning point in AV treatment, is not fully known.

Vitamin A and its metabolites play a complex role in the regulation of insulin sensitivity. While some retinoic acid isomers cause an increase in insulin sensitivity through peroxisome proliferator activating receptors (PPARs), isotretinoin increases the level of total cholesterol and triglyceride and decreases the level of high-density lipoprotein (HDL), resulting in an insulin resistance-like condition (6,7). Controversial results have been obtained about the subject in different studies (8,9).

Recent studies have shown that fat tissue is not only an energy-storing organ, but also has endocrinological and immunological functions. Bioactive mediators secreted by adipose tissue are called adipocytokines (10).

We aimed to evaluate the association of insulin resistance and adipocytokine levels with disease in patients with AV, to explain the metabolic and antiinflammatory effects of isotretinoin treatment on adipocytes, and to evaluate the effects of adipocytokines on insulin resistance development and inflammatory processes during and after isotretinoin treatment.

## 2. Materials and methods

### 2.1. Study group

Twenty-nine patients diagnosed with AV who were older than 18 years of age and had clinical severity of stage II and above were included. The control group consisted of 29 healthy volunteers who did not have any inflammatory skin disease and whose mean age and sex matched with those of the patient group.

People with pregnancy, lactation, hyperlipidemia, liver or renal dysfunction, a systemic disease such as diabetes, atherosclerotic heart disease, thyroid disease, anemia, and metabolic disease were not included in the study. Those with hypervitaminosis, psychiatric disease, muscle disease, and a history of isotretinoin hypersensitivity were not included in the study either. Those who used drugs that interact with isotretinoin such as vitamin A, tetracycline, minocycline were also excluded. Patients with excessive side effects due to current treatment during the treatment period and those without complete remission at the end of the treatment were also excluded from the study.

At the beginning of the study, local ethics committee approval was obtained and a written consent form was obtained from all volunteers stating that they were informed about the study.

### 2.2. Methods

Patients with stage I-IVAV that were refractory to other treatments were included in the study and were treated with isotretinoin at a dose of 0.5 mg/kg/day. Acne severity of patients was determined by clinical staging method; stage I: comedonal acne, stage II: mild papulopustular acne, stage III: severe papulopustular acne, stage IV: nodulocytic acne.

In order to perform body composition analysis, a device of TANITA TBF 300 model based on bioimpedance principle was used.

For serum levels of leptin, resistin, retinol binding protein-4 (RBP4), and insuline, samples were taken from the antecubital region in the morning on an empty stomach after 10–12 h fasting once in the control group and before and after treatment in the patient group. LDL cholesterol, total cholesterol, triglyceride, HDL cholesterol, and fasting blood glucose levels were evaluated and HOMA-IR index (glucose X insulin / 405) was calculated. Body weight, body mass index (BMI), and body fat percentage values ​​were also recorded simultaneously.

Leptin, resistin, adiponectin, and RBP4 were evaluated quantitatively by ELISA in serum samples. In the leptin assay, the DIAsource leptin ELISA (catalog no. KAP2281), the adiponectin assay BioVendor human adiponectin ELISA (catalog no. RD191023100), the resistin assay BioVendor human resistin ELISA (catalog no. RD191016100), and the RBP4 Immunodiagnostik ELISA (K6110) prepared kits were used. At the end of the study, the optical densities formed on the plates were evaluated spectrophotometrically by a 450 nm automated ELISA reader.

### 2.3. Statistical analysis

The analysis of the data was done in SPSS for Windows 11.5 package program. The Kolmogorov–Smirnov test was used to determine whether the distribution of continuous and intermittent variables was appropriate for normal distribution. The significance of differences between the groups in terms of mean values ​​was determined using the Student’s t-test. The significance of the median values ​​was examined using the Mann–Whitney U test. Nominal variables were assessed using the Pearson’s chi-square test. The significance of the difference between the pretreatment and posttreatment clinical measures in the study group in terms of the mean values ​​was assessed using the Wilcoxon sign test, which is significant in terms of median values ​​with the dependent t-test. Whether there is a statistically significant relationship between continuous and intermittent numerical variables was examined using the Spearman’s correlation test. The results for all P < 0.05 were considered statistically significant unless otherwise stated.

## 3. Results

In our study, there were 29 AV patients with 14 (48.3%) female and 15 (51.7%) male AV patients and 16 (55.2%) female and 13 (44.8%) male patients in the control group. There were no statistically significant differences concerning age (P = 0.599) and ratio of the sex (P = 0.131). Acne severity in patients was stage II in 2 (6.9%), stage III in 23 (79.3%), and stage IV in 4 (13.8%) patients (Table 1). 

**Table 1:** The demographic and clinical findings of our study population.

Parameters	Study group	Control group	P-value
Age (year)	20.5 ± 1.9	21.4 ± 2.5	0.131†
Sex (n (%))			
Female	14 (48.3)	16 (55.2)	0.599‡
Male	15 (51.7)	13 (44.8)	
Stage (n (%))			-
II	2 (6.9)	-	
III	23 (79.3)	-	
IV	4 (13.8)	-	

### 3.1. Pretreatment evaluation

There was no statistically significant difference between the patient group and the control group in terms of the mean BMI (P = 0.780), body fat mass (BFM) (P = 0.829), total cholesterol, LDL cholesterol, HDL cholesterol and triglyceride levels before treatment. There were no statistically significant difference between the patient and control groups in terms of median HOMA-IR index (P = 0.182), leptin (P = 0.683), adiponectin (P = 0.978), resistin (P = 0.515), and RBP4 (P = 0.681) levels (Table 2).

**Table 2:** The results of biochemical parameters in our study population.

Parameters	Study group	Control group	P-value
BMI	22.1 ± 2.6	21.9 ± 2.7	0.780†
Body fat mass (kg)	11.7 ± 4.4	11.4 ± 5.0	0.829†
Total cholesterol (mg/dL)	169.0 ± 33.2	162.8 ± 42.7	0.550†
LDL cholesterol (mg/dL)	103.8 (66.0–174.0)	93.8 (49.2–171.8)	0.400‡
HDL cholesterol (mg/dL)	45.5 (33.0–83.0)	48.0 (32.0–79.0)	0.704‡
Trigliserid (mg/dL)	68.0 (40.0–219.0)	81.0 (38.0–296.0)	0.277‡
HOMA	1.55 (0.58–3.64)	1.43 (0.46–2.75)	0.182‡
Leptin (ng/mL)	1.82 (0.67–9.02)	1.82 (0.30–7.31)	0.683‡
Adiponectin (μg/mL)	12.4 ± 4.36	12.4 ± 4.0	0.978†
Resistin (ng/mL)	10.1 (5.7–20.1)	10.8 (5.0–26.3)	0.515‡
RBP4 (mg/L)	30.7 ± 9.1	32.0 ± 13.9	0.681†

There was no significant correlation between disease stage and HOMA, leptin, adiponectin, resistin, and RBP4 levels in the study group (P > 0.05).

###  posttreatment comparison 3.2. Pretreatment vs. posttreatment comparison

When clinical measurements of patients were evaluated before and after treatment, there was no statistically significant difference in terms of BMI (P = 0.477) and mean body fat mass (P = 0.685). There was a statistically significant increase in mean total cholesterol (P < 0.001), LDL cholesterol (P = 0.002), and triglyceride (P = 0.009), and a statistically significant decrease in HDL cholesterol (P = 0.0028).

Isotretinoin treatment did not affect the median leptin (P = 0.125) and adiponectin (P = 0.113) levels, while a statistically significant increase in median HOMA-IR index was observed after treatment (P = 0.028). There was a statistically significant decrease in median resistin (P = 0.016) and median RBP4 (P = 0.040) after treatment (Table 3).

**Table 3:** The effect of the 5-month treatment of isotretinoin on BMI, body fat mass,cholesterol, adiponectin, resistin, and RBP4 levels in AV patients.

Parameters	Pretreatment	Posttreatment	P-value
BMI	21.9 ± 2.7	21.8 ± 2.5	0.477
Body fat mass (kg)	11.4 ± 5.0	11.3 ± 4.4	0.685
Total cholesterol (mg/dL)	162.8 ± 42.7	187.4 ± 51.3	<0.001
LDL cholesterol (mg/dL)	93.8 (49.2–171.8)	122.4 (47.8–189.0)	0.002
HDL cholesterol(mg/dL)	48.0 (32.0–79.0)	44.0 (28.0–73.0)	0.028
Triglyceride (mg/dL)	81.0 (38.0–296.0)	117.0 (47.0–310.0)	0.009
HOMA-IR	1.43 (0.46–2.75)	1.54 (0.57–4.40)	0.028
Leptin (ng/mL)	1.82 (0.30–7.31)	1.93 (0.60–5.04)	0.125
Adiponectin (μg/mL)	12.4 ± 4.0	13.3 ± 4.7	0.113
Resistin (ng/mL)	10.8 (5.0–26.3)	7.1 (5.2–23.9)	0.016
RBP4 (mg/L)	32.0 ± 13.9	36.4 ± 15.8	0.040

In the present study, there was no statistically significant correlation between age, basal BMI, body fat mass, and lipid levels and the amounts of HOMA, leptin, adiponectin, resistin, and RBP4 after treatment (P > 0.025).

## 4. Discussion

Isotretinoin is a vitamin A (retinol) metabolite that has been used for about 30 years in AV treatment (5), but there are few studies addressing the metabolic effects of isotretinoin on humans. Our study showed that isotretinoin treatment resulted in 1) a statistically significant decrease in the mean HDL cholesterol level, 2) increased total cholesterol, LDL cholesterol, and triglyceride levels, 3) increased HOMA-IR value, 4) decreased the mean resistin level, 5) increased the mean RBP4, 6) did not affect the mean leptin level, and 7) no statistically significant correlation between age, pre-treatment BMI, body fat mass, and lipid levels and the amount of change in RBP4 level. These data suggest that isotretinoin therapy causes an increase in insulin resistance independent of differences in age, BMI, body fat, and lipid levels in AV patients.

In our study, in accordance with the literature (8,11–13), total cholesterol, LDL cholesterol, triglyceride levels, and mean HDL cholesterol levels decreased with isotretinoin treatment. Pre- HOMA-IR values ​​that were not different from those of the control group were increased significantly after treatment (P = 0.028) (Table 3). Vitamin A and its metabolites play a complex role in the regulation of insulin sensitivity. While some retinoic acid isomers cause an increase in insulin sensitivity through PPARs, isotretinoin, a vitamin A derivative, cause an insulin resistance-like condition (7). Studies evaluating the effects of retinoid therapy on insulin resistance and glucose metabolism in the literature have yielded different results (6–9,14,15) (Table 4). Koistinen et al. using a hyperinsulinemic-euglycemic clamp technique, found that insulin sensitivity was reduced in 11 patients treated with isotretinoin for 5 months and that a condition similar to insulin resistance syndrome developed with isotretinoin (8). Using the same method, the same group also showed a decrease in insulin sensitivity (7). Heliövaara et al. evaluated insulin resistance by oral glucose tolerance test and reported that isotretinoin causes impaired glucose tolerance (6). In the literature, there have been reports of insulin-dependent diabetes mellitus developed after isotretinoin treatment (16, 17). Corbetta et al. reported a temporary and mild decrease in insulin sensitivity assessed by HOMA-IR in all 10 patients treated with acitretin for only a month (18). These studies, although not explaining the molecular mechanism of this effect, point out that oral isotretinoin affects and reduces insulin sensitivity (6–8), suggesting that isotretinoin may be related to the transcription of various metabolic genes via RAR and RXR (8,19). There are also reports stating that isotretinoin does not affect insulin sensitivity (9,14,15). Ertuğrul et al. (9) in 48 patients and Karadag et al. (15) in 33 patients did not observe a change in HOMA-IR value in the third month of isotretinoin treatment.

**Table 4:** 

Study	Year	Patient number	Retinoid	Time	Results
Koistinen et al. (8)	2001	11M	Isotretinoin	5th month	Insulin sensitivity↓( hyperinsulinemic-euglycemic clamp)
Ertuğrul et al. (9)	2011	48 (35F,13M)	Isotretinoin	3rd month	Insulin sensitivity↔(HOMA-IR)
Stoll et al. (14)	2004	15M	Isotretinoin	5th day	Insulin sensitivity↔(HOMA-IR)
Koistinen et al. (7)	2006	11M	Isotretinoin	5th month	Insulin sensitivity↓(hyperinsulinemic-euglycemic clamp)Adiponectin↑
Corbetta et al. (18)	2006	10M	Asitretin	1st and 3rd month	Insulin sensitivity↓(HOMA-IR)Adiponectin↔Resistin↓
Heliövaara et al. (6)	2007	23 (11F, 12M)	Isotretinoin	3rd month	Insulin sensitivity↓(Oral glucose tolerance test)Adiponectin↑
Karadag et al. (15)	2015	33 (18F, 15M)	Isotretinoin	3rd month	Insulin sensitivity↔(HOMA-IR)Adiponectin↑Leptin↑RBP4↔
Our study		29 (14F, 15M)	Isotretinoin	5th month	Insulin sensitivity↓(HOMA-IR)Adiponectin↔Leptin↔Resistin↓RBP4↑

The results of our study showed that isotretinoin treatment caused an increase in insulin resistance independent of changes in age, BMI, body fat, and lipid levels. The lack of correlation between the increase in lipids and HOMA-IR suggests that the insulin resistance in our AV patients has developed through a different mechanism.

 Recent studies have shown that fat tissue is not only an energy-storing organ, but also has endocrinological and immunological functions. Bioactive mediators secreted by adipose tissue are called adipocytokines (10). Adiponectin, pre-B cell enhancing factor/ visfatin, leptin, RBP4, TNF-α, IL-6, MCP-1, monocyte chemotactic protein-1, and IL-1 are among these adipokines (20). Adiponectin is the most abundant adipocyte in the circulation. This adipocytokine, which increases insulin sensitivity, is also thought to have antiinflammatory effects. Serum concentrations have been shown to decrease in conditions that lead to insulin resistance. Serum level of this cytokine, which is inversely related to BMI, is considered a marker of systemic insulin sensitivity (21).

It has been suggested that isotretinoin causes an insulin resistance-like condition in AV patients (6–8). Adiponectin levels were evaluated before and after treatment in patients treated with isotretinoin, considering that adiponectin is a marker of insulin sensitivity and an antiinflammatory cytokine in our study. In our study, the mean of posttreatment adiponectin levels measured was higher than that of the pretreatment levels, but the difference was not statistically significant (P = 0.113) (Table 3). The literature shows conflicting results about this issue (Table 4). Koistinen et al. evaluated insulin resistance and adiponectin levels in patients prior to treatment, at the 5th month of treatment, and 1 month after termination of treatment, and similar to our study, they found a 34% increase in adiponectin levels compared with the decrease in insulin sensitivity in patients during treatment. In this study, it was suggested that increased adiponectin level despite presence of an insulin resistance may be a physiological adaptation mechanism against insulin resistance (7). Similarly, in a study conducted by Heliövaara et al. (6) with 27 AV patients, it was suggested that isotretinoin increases insulin resistance in patients, paradoxically also increases adiponectin levels and regulates inflammatory parameters. The paradoxical increase of adiponectin was attributed to the antiinflammatory effect of retinoids in the study (6).

Karadag et al. (15) showed that adiponectin levels increased statistically compared to the pretreatment levels in the third month of treatment with isotretinoin in patients with AV, without any evidence of insulin resistance. Transcription of PPARγ coassociated with RXR is increased by treatment with isotretinoin (22). PPARγ has been reported to increase adiponectin gene expression (23). Thus, it has been suggested in a study that prolonged 13-cis retinoic acid treatment may lead to an increase in adiponectin levels by increasing PPARγ transcription (22). Aydin et al. found that basal adiponectin levels of 18 female patients with severe acne were significantly lower than those of the the controls and after 6 months of treatment with isotretinoin, basal adiponectin levels significantly increased without insulin resistance. They revealed increased adiponectin response to oral glucose tolerance test at 2 h in acne patients but this effect was lost after isotretinoin treatment. In this study, it was suggested that isotretinoin treatment restores dysregulation of adiponectin dynamics in severe acne (24).

Contrary to these studies, Zhang et al. showed that a single dose of retinoic acid caused a decrease in adiponectin mRNA levels in adipose tissue of rats (25). Different retinoid derivatives have probably different effects on adiponectin metabolism. Cisneros et al. did not detect any changes in serum adiponectin and leptin levels in rats given low-dose isotretinoin and all-trans-retinoic acid for seven days (5). Mcllroy et al. reported a decrease in adipose tissue adiponectin mRNA levels following a high-fat diet in rats and that this reduction was returned to normal again after treatment with fenretinide, a synthetic retinoid (26). Corbetta et al. reported that there was no change in adiponectin levels in patients with psoriasis while there was a slight decrease in insulin sensitivity and a decrease in resistin levels after one month of treatment with acitretin (18). 

 In the light of these data, a decrease in the adiponectin level was expected in the case of insulin resistance, whereas an increase adiponectin level was observed after isotretinoin treatment in our study, though it was not statistically significant. This can be attributed to the reduction of inflammation by treatment, as noted in similar studies in the literature (23,25). 

In our study, isotretinoin treatment increased the mean leptin level but the difference was not enough to produce a statistically significant effect (P = 0.125) (Table 3). Leptin is a cytokine-like hormone which is a gene product of obese (ob) and is mostly synthesized by subcutaneous adipose tissue. The level correlates with the amount of BMI and adipose tissue (21). The main task of leptin is to regulate body weight and appetite (27). For this reason, this cytokine is closely related to insulin and glucose metabolism. Leptin has also proinflammatory effects and is involved in natural and acquired immunity. In the literature, inhibition of leptin secretion has been shown to be induced by retinoic acid derivatives in animal studies in which retinoic acid derivatives other than isotretinoin were used. In our study, no change in leptin levels after long-term isotretinoin denies any effect of isotretinoin on leptin levels in AV patients.

Resistin is an adiponectin produced by adipocytes, adipose tissue monocytes, macrophages, and peripheral blood monocytes. In addition to their role in adipogenesis, inflammation, and cardiovascular disease, many studies (21,28,29), but not all (30,31), show increased levels of plasma/tissue in obesity and diseases associated with insulin resistance. In our study, isotretinoin treatment for 5 months reduced the mean resistin level (P = 0.016) in 29 AV patients (Table 3). On the other hand, there was no correlation between the stage of the disease and the pretreatment and postreatment resistin levels and there was no statistically significant correlation between age, pre-treatment BMI, body fat mass, and lipid levels and the amount of change in resistin level with treatment. Felipe et al. suggested that thiazolidinediones in the antidiabetic drug group, which have inhibitor effect on the resistin gene, act through PPARgamma and that PPARgamma coexists with RXR; thus, retinoic acid may affect resistin expression. In the study, it was reported that resistin expression in rats that were given 9-cis retinoic acid and all-trans retinoic acid was suppressed, and tissue and blood levels of resistin were decreased (32).

Similar to our results in acne patients, Corbetta et al. have reported that acitretin in psoriasis patients caused a slight decrease in insulin sensitivity and a decrease in resistin levels but did not affect adiponectin levels in psoriatic patients and excluded the role of adipocytokines in retinoid-induced insulin resistance (18) (Table 4). Supporting their study, we have found a reduction in resistin levels, despite the finding indicating that a five-month treatment of isotretinoin is related with insulin resistance in our study. No other parameter was shown to correlate with the change in resistin levels. Retinoids may be involved in mechansims in decreasing resistin levels directly by regulating resistin expression independently of these parameters.

RBP4 is a recently defined protein that binds to circulating retinol. RBP4 is thought be related to obesity, insulin resistance, type-2 diabetes, and metabolic syndrome (33) and is suggested to be an early indicator of the development of insulin resistance and metabolic syndrome, and that this adiponectin is more sensitive to insulin than leptin, IL-6, and CRP (34). Our study suggests that a 5-month treatment with isotretinoin increases the mean RBP4 level in AV patients (P = 0.040) (Table 3). However, there was no significant correlation between the disease stage and pretreatment and posttreatment RBP4 levels. In addition, no significant correlation was observed between age, pretreatment BMI, body fat mass, and lipid levels, and the amount of change in RBP4 levels. Koistinen et al. suggested that isotretinoin taken for 5 months caused insulin resistance in AV patients, which was thought to be related to RBP4 (7). Another study indicated that isotretinoin treatment for three months did not affect RBP4 levels (15) (Table 4). Considering the sensitivity of RBP4 to show the insulin resistance, our study points out that long-term treatment with isotretinoin increases the insulin resistance in patients with AV.

In conclusion, our study suggests that isotretinoin therapy in AV patients causes insulin resistance in these patients regardless of age, BMI, body fat mass, and lipid levels of these AV patients. Our study confirmed the effect of isotretinoin on insulin resistance but lacked to point out the parameters that affect the mechanism of this effect. The limitations of our study are the low number of patients and lack of long-term follow-up of patients after treatment.
